# Supplementary material for: Isomelezitose Overproduction by Alginate-Entrapped Recombinant E. coli Cells and In Vitro Evaluation of Its Potential Prebiotic Effect
Source: Int J Mol Sci. 2022 Oct 21;23(20):12682. doi: 10.3390/ijms232012682 (PMC9604484; doi:10.3390/ijms232012682)
Supplement: Supplementary file 1 [file ijms-23-12682-s001.zip › ijms-1954375-supplementary.pdf]

SUPPLEMENTARY MATERIAL

Isomelezitose overproduction by alginate-entrapped recombinant *E. coli* cells and in-vitro evaluation of its potential prebiotic effect

Martin Garcia-Gonzalez[1], Fadia V. Cervantes[2], Ricardo P. Ipiales[3], Angeles de la Rubia[3], Francisco J. Plou[2], and Maria Fernandez-Lobato[1]*

[1] Departamento de Biología Molecular, Instituto de Biología Molecular, Centro de Biología Molecular Severo Ochoa (CSIC-UAM), Universidad Autónoma de Madrid. C/ Nicolás Cabrera, 1, 28049 Madrid, Spain

[2] Instituto de Catálisis y Petroleoquímica, CSIC, 28049 Madrid, Spain

[3] Departamento de Ingenería Química, Universidad Autónoma de Madrid (UAM). Campus de Cantoblanco 28049 Madrid, Spain

* Corresponding author: Maria Fernandez-Lobato (mfernandez@cbm.csic.es)

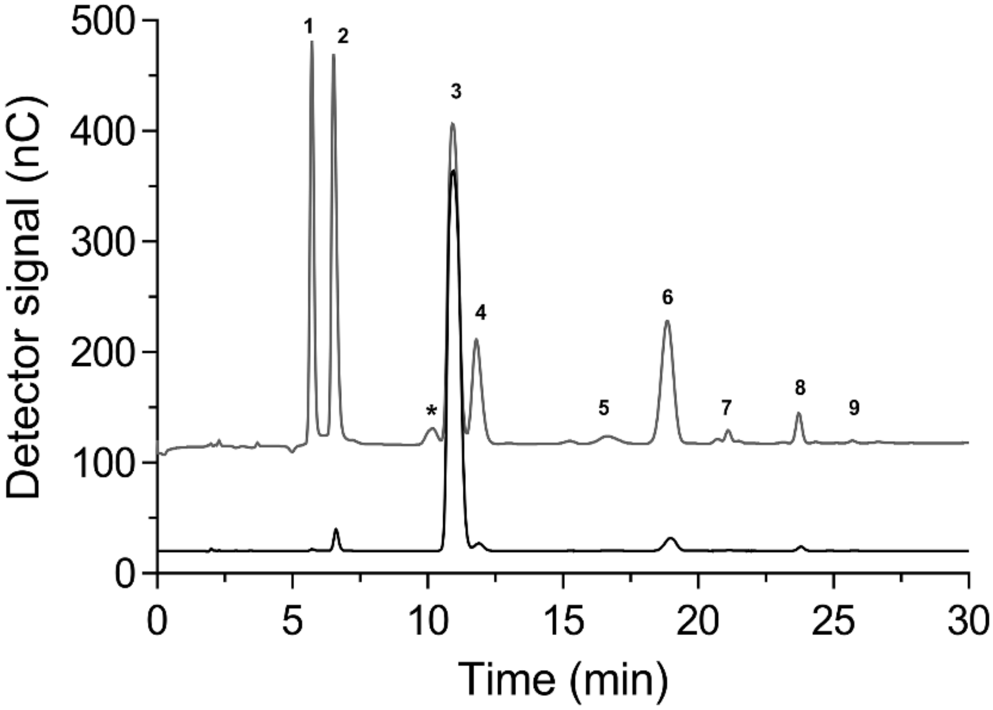

Figure S1. Analysis of hetero-GlcOS production catalysed by alginate-entrapped *E. coli* [Mr*α*Glu-pET28b(+)] cells. HPAEC-PAD chromatograms of the reactions performed with not induced immobilized *E. coli* cells (black line) and with 1 mM IPTG induced cells (grey lines) are shown. Alginate-based biocatalyst with cells that were not induced with IPTG practically did not hydrolysed sucrose and so, the peaks corresponding to the transglucosylation products were negligible. Peak assignation: **(1)** D-glucose; **(2)** D-fructose; **(3)** sucrose; **(4)** trehalulose; **(5)** melezitose; **(6)** isomelezitose; **(7)** theanderose; **(8)** erlose; **(9)** esculosa; **(*)** unknown product.

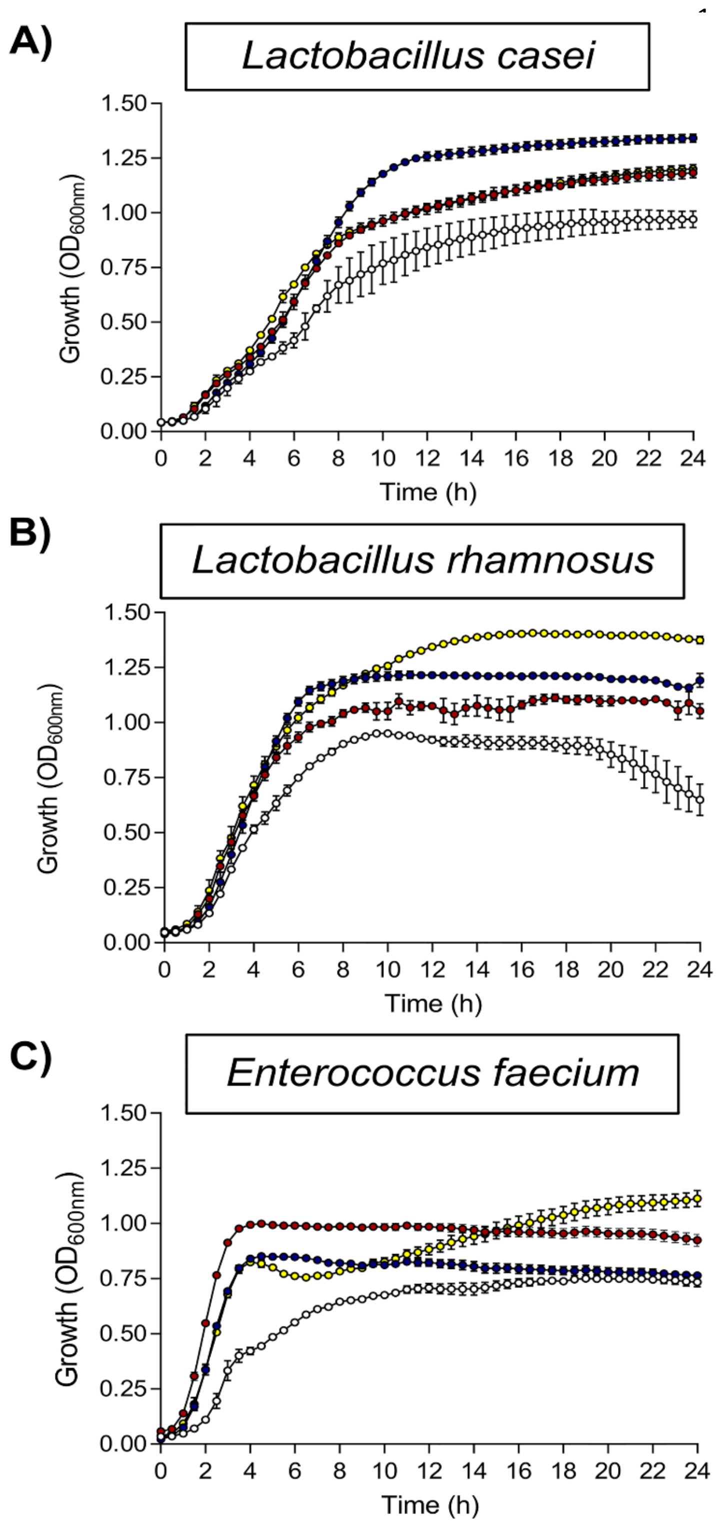

Figure S2. Growth curves of the indicated probiotic bacteria. Bacteria were cultured at 37 ºC and under aerobic conditions in MRS medium with no carbon source (white circles), or supplemented with glucose, fructose and sucrose at the same concentration in the hetero-GlcOS mixture (yellow circles), 2.0% (w/v) "Actilight" FOS mixture (blue circles) or 2.0% (w/v) hetero-GlcOS mixture (green circles). Error bars represent the standard deviations from three independent analysis.

Table S1. Sugar composition of the reaction mixture treated with alginate-entrapped *Komagataella phaffii* cells.

Carbohydrate (% w/w)	Glucose	Fructose	Sucrose	Trehalulose	Isomelezitose	Rest of hetero-GlcOS
**Before**	18	25.5	13	19	15	9.5
**After**	1.2	0.6	22	33	26	17.2

Reaction conditions: 30 h incubation with orbital shaking (200-250 rpm) at 30 ºC.

Table S2. Composition of the commercial fructo-oligosaccharides mixture "Actilight" determined by HPAEC-PAD.

Carbohydrate	Concentration (% w/w)
**Glucose**	0.4
**Fructose**	0.7
**Sucrose**	11.1
**1-Kestose**	33.0
**Neokestose**	2.3
**Nystose**	35.6
**Neonystose**	4.2
**1-Fructosylnystose**	7.3
**1-Fructosylfructosylnystose**	3.3
**Other FOS**	2.2

The HPAEC-PAD method is described in Section 3.10 of *Materials and Methods*.
